# Evaluation of a Particle Enhanced Turbidimetric Immunoassay (PETIA) for detecting calprotectin in sputum samples

**DOI:** 10.1186/s12890-026-04195-1

**Published:** 2026-02-21

**Authors:** Magnus Förnvik Jonsson, Ulrik Hägglund, My Eltén, Torgny Sunnerhagen, Muna Al-Jammal, Magnus Paulsson

**Affiliations:** 1 Section for Clinical Chemistry, Department of Translational Medicine, Lund University, Malmö, Sweden; 2 Department of Clinical Chemistry and Pharmacology, Skåne University Hospital, Lund, Sweden; 3 Department of Clinical General Laboratory Medicine, Skåne University Hospital, Malmö, Sweden; 4 Division of Infection Medicine, Department of Clinical Sciences, Lund University, Lund, Sweden; 5 Department of Clinical Microbiology, Skåne University Hospital, Lund, Sweden

**Keywords:** Airway inflammation, Calprotectin, COPD, Cystic fibrosis, Sputum, Stability, Turbidimetric, PETIA

## Abstract

**Background:**

Chronic obstructive pulmonary disease (COPD) and cystic fibrosis (CF) are characterized by persistent airway inflammation and airflow limitation. Measuring airway inflammation may be of value both in research and clinical practice. Calprotectin, a protein associated with neutrophil activation, serves as a potential biomarker for airway inflammation. This study evaluates the performance of a particle enhanced turbidimetric immunoassay (PETIA) for measuring calprotectin in sputum samples.

**Methods:**

Thirty sputum samples from unique patients were collected at the Department of Clinical Microbiology in Lund, Sweden and treated with dithiothreitol (DTT) before analysis. Calprotectin levels were measured using a PETIA on an automated turbidimetric analysis system (Atellica CH 930 Analyzer). The 30 samples were analyzed in triplicate the day of sample collection (Day 0), after storage at 4 °C for two to four days (Day 2–4), and after six to seven days (Day 6–7). An additional aliquot from each sample was stored at -20 °C and analyzed after 41 to 58 days (Day 41–58). Precision, accuracy, and stability of the assay were assessed. Precision was evaluated using pooled- and individual sputum samples while accuracy was determined through spiking studies and dilution linearity. Stability was tested by comparing calprotectin levels Day 2–4, Day 6–7 and Day 41–58 with Day 0, respectively. Instability equations were calculated for samples stored at 4 °C and at -20 °C, respectively.

**Results:**

Three different pools were each analyzed 25 times during five days. The coefficient of variation (CV) for the calprotectin concentration was below 3% for all three. Considering precision based on individual samples, 90% of 120 triplicates had a CV below 7%. These figures indicate an acceptable precision. The spiking and dilution studies showed that measured calprotectin concentrations matched expected levels, with deviations below 10%. Calprotectin concentrations Day 2–4, Day 6–7 and Day 41–58 did not significantly differ from Day 0, and the slope of the instability equations did not significantly differ from 0. It implies that calprotectin concentration can be measured in DTT treated sputum stored for up to one week at 4 °C and up to two months at -20 °C.

**Conclusion:**

The evaluated PETIA, running on an automated system, provides a promising method for measuring calprotectin in sputum, supporting its potential use in both clinical and research applications.

## Background

Chronic obstructive pulmonary disease (COPD) and cystic fibrosis (CF) are associated with airway inflammation and airflow limitation [1]. Measurements of inflammation biomarkers in COPD and CF have multiple potential applications in research and clinical practice, including monitoring of inflammation, predicting exacerbations and assessing treatment response [2–4]. Expectorated sputum samples reflect the intrabronchial content of the lower respiratory tract and are routinely collected for microbiological tests and cultures. However, sputum also contains proteins related to bronchial or pulmonary inflammation. Since inflammatory processes are compartmentalized, measurements of inflammatory mediators in sputum are potentially valuable biomarkers of pulmonary inflammation. However, they remain underutilized in clinical practice due to limited standardization and insufficient scientifical validation [4, 5].

Calprotectin is a 24 kDa heterodimer and member of the S100 protein family. It is composed of two calcium-binding proteins, S100A8 and S100A9, and is expressed in multiple tissues and cell types. It is present in high concentrations in the cytosol of neutrophils and released into the extracellular space upon degranulation together with other effector proteins and neutrophils extracellular traps [6–8]. Hence, calprotectin concentration in sputum can be regarded as a marker of neutrophil activation. The protein is associated with several biological functions, including proinflammatory properties that lead to cytokine secretion and leukocyte recruitment and modulation of macrophage functions [9]. There is a strong correlation between the level of calprotectin in plasma and the degree of inflammation in several acute and chronic diseases [10, 11].

Despite calprotectin being a promising biomarker for monitoring airway inflammation [12–15] data on robust assays are limited, especially ones adapted for respiratory samples which are suitable for automation in hospital laboratories. Commercially available turbidimetric calprotectin immunoassays developed for automated platforms are typically validated for serum or plasma samples. Sputum is a different and complex matrix and require pretreatment with a reducing and mycolytic solution containing dithiothreitol (DTT), making separate studies of calprotectin measurement in sputum necessary [16].

To enable clinical use of calprotectin measurements in sputum samples we have evaluated analytical performance on sputum for an assay with proven good performance on serum/plasma and developed for automated turbidimetric analysis. We have studied precision and accuracy for calprotectin concentration measurements, as well as stability for sputum samples stored up to one week in fridge and up to two months in freezer, respectively.

## Methods

### Study population and sample collection

Thirty sputum samples from unique patients that were sent for bacterial cultures to the Clinical Microbiology department, Region Skåne, Lund, Sweden during October 2024, were identified and included in this study. Inclusion criteria were: (1) samples were sent for general bacterial cultures and (2) the volume of the sample after microbial tests was sufficient for the planned analyses. No detailed clinical information (e.g., diagnosis, spirometry, smoking status) was collected, as the study focused solely on analytical performance.

### Preanalytical sample handling

To reduce viscosity and improve homogeneity of the samples, equal volumes (at least 1mL) of sputum and working solution of DTT (Sputolysin, Merck KGaA, Darmstadt, Germany) were mixed by vortexing and incubated 15 min at room temperature. The DTT concentration in the incubated solution was 3.25 mM DTT. Thereafter a 10-fold dilution in sterile saline was prepared in a new tube resulting in a final DTT concentration of 0.33 mM. The diluted solution was transported the same day cooled to the analytical unit where it was centrifuged at 2000 x g for 10 min. Hereafter, 1 mL of the supernatant was transferred to a new tube and stored at 4 °C, and 1 mL of the supernatant was transferred to another tube and stored at -20 °C. All refrigerators and freezers used for sample storage were continuously temperature‑monitored and alarm‑connected, in accordance with the laboratory’s quality management system (SS‑EN ISO 15189:2012). Patient samples and pools were analyzed and stored in 5 mL tubes (REF 55.476, Sarstedt AG & Co. KG, Nümbrecht, Germany). These tubes were also used when preparing and analyzing samples in the in the spiking experiments. Samples to evaluate linearity were prepared and analyzed in 1 mL tube-top samples cups (REF 11069061, Siemens Healthiners, Erlangen, Germany).

### Calprotectin assay

Calprotectin concentration measurements were done on an Atellica CH 930 Analyzer (Siemens Healthineers) with reagents and calibrator kit from Gentian Diagnostics (Moss, Norway) according to instructions for use from the manufacturer [17]. It is a particle enhanced turbidimetric immunoassay (PETIA). The assay is used in routine analysis for plasma samples, and when verifying the assay for plasma the Gentian Calprotectin kit controls were analyzed 25 times during 5 days resulting in a CV of 4% at a level of 1 mg/L and a CV of 2% at a level of 10 mg/L (since no preanalytical treatment and dilutions were applied to the kit controls these levels correspond to 20 mg/L and 200 mg/L calprotectin, respectively, in sputum samples). During a 3-month period in routine analysis the same controls showed CVs of ~ 6% and ~ 4%, respectively. At each analysis session, those kit controls at 2 levels were used to verify that the assay worked as intended. Kit-controls were handled as other controls on the Atellica instrument and stored cooled onboard. Patient samples and pools were kept at a temperature between 20 °C and 25 °C for at least 30 min and thereafter mixed thoroughly before analysis. Stored samples were also centrifugated at 2000 x g for 10 min just before measurement. Each individual sputum sample was analyzed in triplicate.

The detection range according to the manufacturer is ~ 0.4–20 mg/L [17]. If the concentration exceeded the measuring range, the sample was automatically diluted 5-fold in CH-diluent (Siemens Healthineers) by the instrument. Including the preanalytical dilution (20x), the measuring range in sputum was ~ 8-400 mg/L and the extended range ends at 2000 mg/L.

### Precision of sputum measurements

Three pools (low, medium, and high concentration) were created by pooling patient sputum samples. Each pool was analyzed for five consecutive days with five measurements per day. Measurements were done on the same instrument, calibration, and reagent lot. Imprecision expressed as coefficient of variation (CV) based on all 25 measurements was calculated for each pool. Individual sputum samples were analyzed in triplicate as part of the stability study (see below). These measurements were also used to approximate the imprecision by calculating CV for each triplicate. Acceptable CV for individual sputum samples was considered to be below 20% [16, 18].

### Accuracy of sputum measurements

To evaluate the effect of the preanalytical treatment with (DTT), two different volumes of a kit-calibrator (with 21.76 mg/L calprotectin) were spiked into diluted solutions of Sputolysin and incubated for 15 min. The two incubated mixtures contained 10.88 mg/L and 16.32 mg/L calprotectin, respectively, in presence of 3.25 mM DTT (the same concentration as patient samples were incubated in). Thereafter, both solutions were diluted 10-fold in saline resulting in a theoretically expected calprotectin concentration of 1.09 and 1.63 mg/L, respectively, in presence of 0.33 mM DTT. A control was made for each calprotectin level by spiking the kit-calibrator into phosphate buffered saline resulting in a final calprotectin concentration of 1.09 and 1.63 mg/L respectively (in absence of DTT). Calprotectin was measured in triplicate for all four preparations. Recovery was calculated by comparing measured values with expected (theoretically calculated based on amount calibrator added).

To further evaluate the sample matrix, kit-calibrator was spiked into a sputum pool. Sputum samples prepared by the preanalytical procedure above were pooled. Thereafter three mixtures were prepared: (a) 100 µL kit-calibrator solution (2176 ng calprotectin) was added to 400 µL sputum pool, (b) 50 µL kit-calibrator solution (1088 ng calprotectin) and 50 µL saline were added to 400 µL sputum pool and (c) 100 µL saline was added to 400 µL sputum pool. Sputum pool as well as mixtures a-c were measured in triplicate. The concentration in the sputum pool and known amounts of added calprotectin was used to calculate expected concentration in mixtures a-c. Final volume of the mixtures was assumed to be 500 µL. Spiking was evaluated by recovery calculated as measured/expected concentrations for mixtures a-c.

Dilution linearity was also part of the accuracy evaluation. Two different dilution series were created using unique sputum samples. Each series was created by mixing a sample containing a high concentration of calprotectin with a sample containing a low concentration in different proportions. Calprotectin concentrations were measured in triplicate and recovery was calculated by comparing the mean values with expected levels. Acceptable recovery for spiking and dilution studies was considered to be within 80–120% [19].

### Stability of sputum samples

Stability testing was performed using the 30 individual samples. They were analyzed the day of sample collection (Day 0), after storage at 4 °C for two, three or four days (Day 2–4), and after six or seven days (Day 6–7). An additional aliquot from each sample was stored at -20 °C and analyzed after 41 to 58 days (Day 41–58).

The mean of the triplicate measurements done Day 2–4, Day 6–7 and Day 41–58 were compared with measurements performed on day 0 (baseline), respectively. The comparison was statistically evaluated using Passing-Bablok regression. If the 95% confidence interval for the intercept included 0 and for the slope included 1, it indicated no significant difference between the measurements.

Stability was further evaluated according to suggestions from CRESS recommendation [20]. For each sputum sample the percentage difference (PD%) of the concentration measured after storage compared to Day 0 (baseline) was calculated. The mean of the triplicate was used. PD% plotted versus days in refrigerator (4 °C) and least-square-fit (forced through origo) with 95%-confidence interval were used to calculate the instability equation, PD%=slope x time (in days). A separate fitting was done for samples stored in the freezer (-20 °C). PD < 10% according to instability equation when setting time to 7 for samples stored in fridge and 60 for samples stored in the freezer was considered acceptable.

Average PD% and its 95%-confidence interval (1.96 x standard error of mean) for samples stored the same or similar time period was also calculated.

### Statistics

R version 4.5.2 was used to calculate instability equations and corresponding 95%-confidence interval. For all other statistics calculations and for plotting the graphs Microsoft Excel and Analyse-it for Microsoft Excel (version 6.15.4) were used.

### Ethical review

This study was approved by the Swedish Ethical Review Authority (Dnr 2024-03624-01).

## Results

The study included 30 sputum samples from unique patients. The median age was 69.5 years (range 14–91), with 20 men and 10 women.

### Precision of sputum measurements

**Fig. 1:**
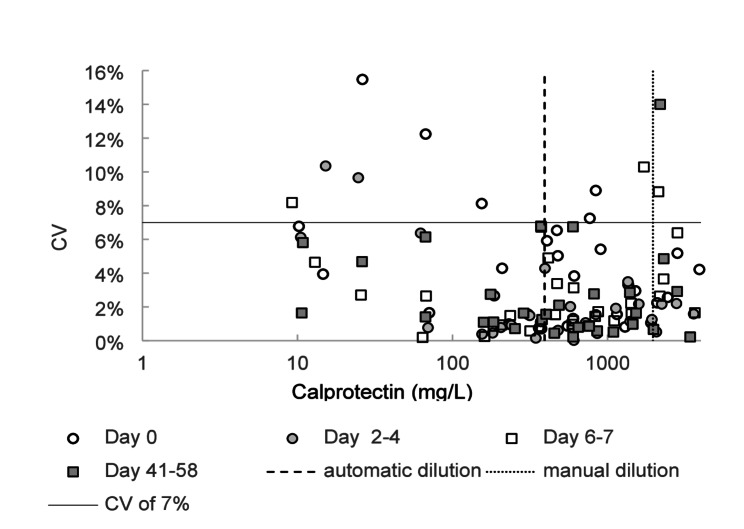
Imprecision based on individual samples. Circles and boxes represent coefficient of variation (CV) from triplicate calprotectin measurements in sets of 30 sputum samples at four different time points: Day 0, Day 2–4, Day 6–7 and Day 41–58. 90% of the triplicates have a CV below 7%, indicated by a solid line

The precision of the calprotectin assay for sputum analysis was assessed by determining the relative variability of repeated measurements using both pooled and individual sputum samples. Repeated measurements of the low/medium/high concentration pools yielded CVs of 1.1% at 58 mg/L, 2.8% at 113 mg/L, and 2.4% at 219 mg/L, respectively. In addition, the 30 individual sputum samples were analyzed in triplicate at four different occasions separated in time as part of the stability test, and CV was calculated for each triplicate set (Fig. 1 and Supplemental Table). Analysis of the variability across all triplicate measurements showed that all had a CV below 16%, 97.5% had a CV below 11%, and 90% had a CV below 7%. As illustrated in Fig. 1, the precision was comparable between fresh and stored samples. Overall, the CV-distribution indicates acceptable precision of the calprotectin assay, regardless of sample concentration range and tested storage conditions.

### Accuracy of sputum measurements

The preanalytical treatment was evaluated by incubating two different concentrations of calibrator with and without DTT, respectively, and thereafter diluting them (the same procedure as for patient samples). Samples with and without DTT gave very similar values and recovery in relation to expected values was between 98 and 100%, see Table 1. Thus, there is no evidence that DTT at concentrations used affect the measurement of calprotectin.

**Table 1:** Calprotectin incubated in presence and absence of DTT

Preparation	Measured conc. in mg/L^a^ (x20)^b^	Recovery in % (measured / expected)
CS^c^ incubated in 3.25 mM DTT for 15 min, thereafter diluted (x10) to 1.09 mg/L calprotecin and 0.33 mM DTT before analysis	1.09 (21.8)	100
CS incubated in absence of DTT for 15 min, thereafter diluted (x10) to 1.09 mg/L calprotecin before analysis	1.08 (21.6)	99
CS incubated in 3.25 mM DTT in 15 min, thereafter diluted (x10) to 1.63 mg/L calprotecin and 0.33 mM DTT before analysis	1.60 (32.0)	98
CS incubated in absence of DTT for 15 min, thereafter diluted (x10) to 1.63 mg/L calprotecin before analysis	1.60 (32.0)	98

^a^Reported calprotectin concentration from the instrument (mean of triplicate)

^b^Preinstrumental dilution factor of 20 included to be able to compare the levels with measurements on individual sputum samples

^c^CS: Calibrator solution

Thereafter, a pool of sputum samples was spiked with two different amounts of calibrator and saline (as a control), respectively. Measured and expected values are presented in Table 2. Recovery was between 99 and 102%. Thus, added calprotectin was found in expected concentration in the spiked samples.

**Table 2:** Spiked sputum pool

Preparation	Measured concentration^a^ in mg/L (x20) ^b^	Expected concentration in mg/L (x20)	Recovery in % (measured / expected)
400 µL Sp. pool ^c^ + 100 µL CS^d^	9.50 (190)	9.59 (192)	99
400 µL Sp. pool + 50 µL CS + 50 µL saline	7.31 (146)	7.42 (148)	99
400 µL Sp. pool + 100 µL saline	5.36 (107)	5.24 (105)	102
Sp.pool	6.55 (131)		

^a^Reported calprotectin concentration from the instrument (mean of triplicate)

^b^Preinstrumental dilution factor of 20 included to be able to compare the levels with measurements on individual sputum samples

^c^Sp. pool: eight individual sputum samples incubated in a sputolysin-solution and further diluted in saline (final dilution of sputum x20) were pooled

^d^CS: kit-calibrator solution containing 21. 76 mg/L calprotecin

Accuracy was further assessed by dilution linearity. Individual sputum samples with high and low calprotectin levels were mixed in defined proportions, and the measured values were compared to expected concentrations. The recovery rates for the mixtures were within 10% of the expected values (Table 3), indicating a good agreement [19]. This demonstrates that calprotectin in sputum samples behaves in a similar way as the assay calibrator when diluted and also supports that calprotectin can be accurately measured in that matrix.

**Table 3:** Recovery of calprotectin in two different dilution series

High (H) & low (L) samples in different proportions	Sample H1 and L1			Sample H2 and L2		
	M (mg/L)	E (mg/L)	*R* (%)	M (mg/L)	E (mg/L)	*R* (%)
100% H	217			266		
80% H, 20% L	181	187	97	234	228	103
60% H, 40% L	152	157	97	193	190	102
40% H, 60% L	126	127	100	164	151	108
20% H, 80% L	100	96	104	123	113	109
100% L	66			74		

Samples with high (H) and low (L) concentrations of calprotectin were mixed in predefined ratios to enable analysis of linearity of the values in diluted samples. The the measured (M) values (mean of triplicates) and expected (E) are presented in mg/L and the recovery (R) in percentage

### Stability of calprotectin in sputum samples

**Fig. 2:**
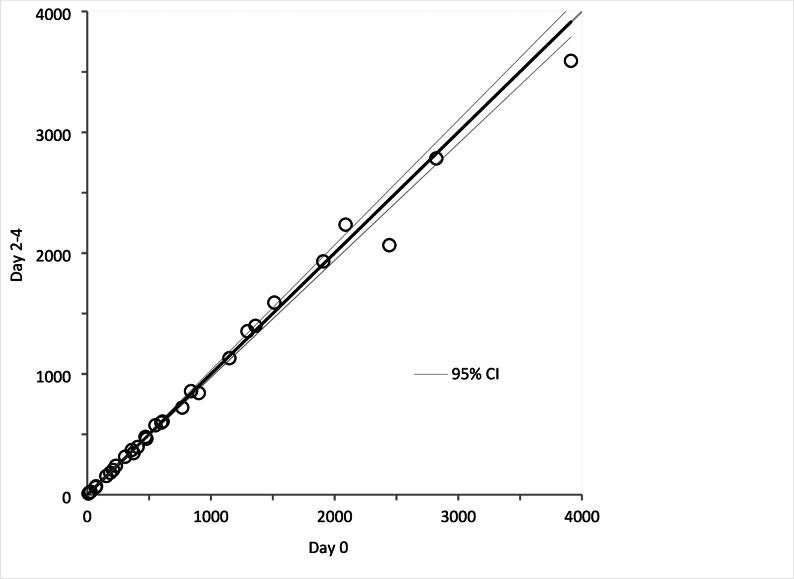
Stability of fresh samples stored in refrigerator for 2 to 4 days. Circles represent calprotectin concentration (mg/L, mean of triplicates) in 30 sputum samples measured Day 0 and Day 2–4, and compared by Passing-Bablok regression analysis. 95% -confidence interval (CI) for intercept and slope were − 7.72 to 5.62 and 0.97 to 1.04, respectively

**Fig. 3:**
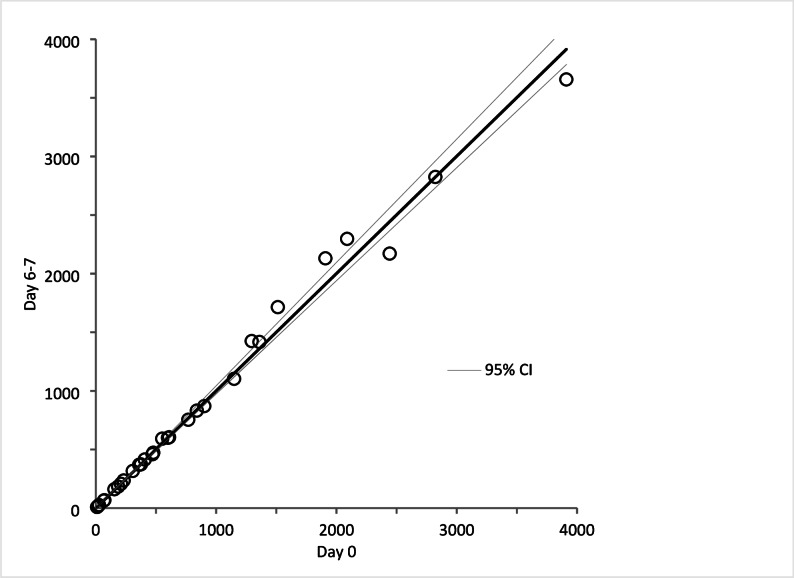
Stability of fresh samples stored in refrigerator for 6 to 7 days. Circles represent calprotectin concentration (mg/L, mean of triplicates) in 30 sputum samples measured Day 0 and Day 6–7, and compared by Passing-Bablok regression analysis. 95%-confidence interval (CI) for intercept and slope were − 8.38 to 10.25 and 0.97 to 1.05, respectively

**Fig. 4:**
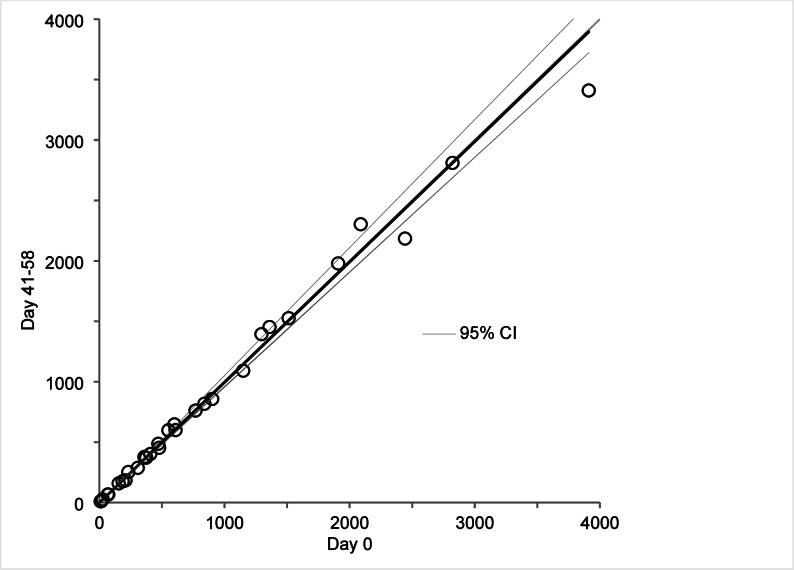
Stability of samples stored in freezer for 41 to 58 days. Circles represent Calprotectin concentration (mean of triplicates) in 30 sputum samples measured Day 0 and Day 41–58, and compared by Passing-Bablok regression analysis. 95%-confidence interval (CI) for intercept and slope were − 13.34 to 11.58 and 0.95 to 1.06 respectively

Stability was evaluated by comparing calprotectin levels in sputum samples stored at 4 °C and − 20 °C over various time intervals. Measurements performed on Day 2–4, Day 6–7, and Day 41–58 were compared to baseline values on Day 0 (Figs. 2, 3 and 4). No significant differences were observed, as the 95% confidence intervals for the regression analysis included 0 for the intercept and 1 for the slope.

**Fig. 5:**
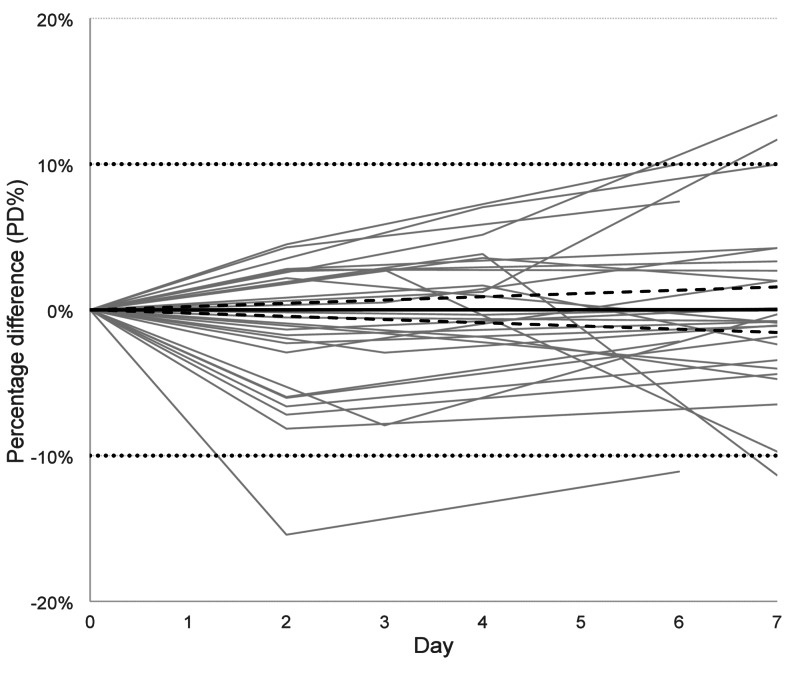
Stability of 30 fresh sputum samples stored in refrigerator for up to 7 days. Each grey line represents a patient sample with calprotectin measured in triplicate at Day 0 (baseline), Day 2–4 and Day 6–7. Percentage difference (PD%) based on mean of triplicates was calculated in relation to Day 0 for all measurements done Day 2–4 and Day 6–7. Instability equation (black line), PD%=0.0017 x time (Days), was calculated based on all data points using least-square-fitting. The 95%-confidence interval for the slope was from − 0.22 to 0.22 (dotted lines). Dotted horizontal lines at ± 10% indicate the predefined acceptance criterion, allowing direct visual assessment of whether the fitted instability trend stays within acceptable limits

PD% was calculated for stored samples and used to obtain instability equations for fresh (Fig. 5) and frozen samples (Table 4), respectively. Numerical data for all individual samples, including triplicate values, CV % and PD %, are provided in Supplemental Table. Using the equation for fresh samples to calculate the 95% confidential interval for PD% at day 7 gave the range − 1.54% to 1.54%, and using the equation for frozen samples at day 60 gave the range − 3.48% to 1.50%. Both intervals pass zero and are far from the limit +/-10%. Summarized, there is no evidence for systematic deviation of the stored samples. These findings indicate that the NaCl-diluted and DTT pretreated sputum preparations remain stable in the refrigerator for at least one week and in the freezer for at least two months.

**Table 4:** Stability data for frozen sputum samples

Calprotectin	Deviation from Day 0 (baseline)								
	Day 41 *n* = 3	Day 42 *n* = 3	Day 43 *n* = 4	Day 44 *n* = 4	Day 47 *n* = 2	Day 48 *n* = 5	Day 49 *n* = 2	Day 56 *n* = 3	Day 58 *n* = 4
PD%,mean (95%-CI)^a^	-7.12% (-25.64%, 11.40%)	-2.90% (-6.28, 0.49%	-7.73% (-13.22, -2.24%	2.80% (-0.74, 6.34)	-1.14% (-8.83, 6.54)	5.10% (-0.56, 10.77)	-3.87% (-6.36, 1.37)	-0.53% (-5.83, 4.76)	1.25% (-7.42, 9.92)

^a^The mean percentage deviation (PD%) and its 95%-confidence interval (CI) for samples stored in the freezer the same number of days. Instability equation based on all data points using least-square-fitting was PD%=-0.016 x time (Days) and 95%-CI for the slope was from − 0.058 to 0.025

## Discussion

This study evaluated the performance of an automated PETIA for measuring calprotectin concentrations in clinical sputum samples. The results demonstrate that the assay has acceptable precision, accuracy and stability.

The assay precision was assessed by analyzing pooled and individual sputum samples. The total CVs observed for the three pools were below 3.0%., indicating high precision under controlled conditions. Notably, these figures are very similar to those seen for kit controls during verification of the assay for plasma samples. However, the CVs based on triplicate measurements from individual samples showed a broader range of values. Although 90% of triplicates had CVs below 7%, the three triplicates with the greatest spread had CVs between 11% and 15%. Differences in CV estimates between pools and individual samples are not uncommon and may indicate that matrix effects differing between samples may influence the performance of the assay. This is an expected finding considering the inherent heterogeneity and complexity of sputum as a biological matrix, despite pretreatment with DTT intended to reduce this variability [12]. The overall assessment is that precision is acceptable across the entire measuring range of the assay [16, 18].

The accuracy of the assay was validated through spiking and dilution linearity experiments. One part of the spinking tests was designed to evaluate the preinstrumental handling. The close agreement between the measured and expected calprotectin concentrations in all spiked and diluted samples suggests that the assay accurately quantifies calprotectin levels in sputum samples handled according to our preanalytical protocol. This is important for being able to introduce the analysis in clinical practice, where accurate measurements enable diagnosing and monitoring of airway inflammation.

The stability tests demonstrated that calprotectin levels in DTT-treated sputum samples remain consistent over time when stored at 4 °C for up to one week and at -20 °C for at least two months. This stability is advantageous for clinical laboratories, as it allows for flexible sample handling and storage without compromising the integrity of the measurements.

While this study provides valuable insights into the performance of the PETIA for calprotectin detection in sputum, there are some important limitations to consider. Only one Sputolysin preparation was done on each sputum sample, so the full preanalytical variation cannot be estimated given the study design. The study was designed as a methodological validation and therefore did not include detailed clinical data such as diagnosis, spirometry, smoking status, sputum cellular composition, or bacterial load. Consequently, no correlation analyses with disease severity or microbiological findings were performed. Although the collected sputa all had measurable levels of calprotectin, the relative small number of samples and not knowing the clinical condition of the patient when the sputum was collected makes it difficult to know what concentrations to expect at different conditions and assess whether this method can measure low enough levels or if there is a substantial risk for antigen excess in a clinical setting. All these aspects were beyond the scope of this work but will be addressed in future studies with larger, well-characterized cohorts to evaluate clinical applicability across different respiratory conditions, including COPD, CF, asthma, and lower respiratory tract infections. Potentially, a more personalized approach to managing respiratory conditions is possible by utilizing sputum calprotectin levels as a biomarker for airway inflammation. Regular monitoring of calprotectin levels can potentially help predict exacerbations, guide antibiotic therapy, and assess treatment response, ultimately improving patient outcomes.

## Conclusions

The automated PETIA for calprotectin detection in sputum samples demonstrates adequate precision, accuracy and stability. In addition, assay performance was maintained in samples stored at 4 °C for up to one week and at -20 °C for up to two months, increasing flexibility by allowing delayed analysis on stored samples without compromising reliability. These findings support the PETIA as a tool for clinical and research applications, enabling the potential use of sputum calprotectin as a biomarker for airway inflammation which holds promise for improving the diagnosis and management of both acute and chronic respiratory conditions.

## Data Availability

The datasets used and/or analyzed during the current study are available from the corresponding author on reasonable request.

## Abbreviations

COPD: Chronic obstructive pulmonary disease
CF: Cystic fibrosis
DT: Dithiothreitol
PETIA: Particle enhanced turbidimetric immunoassay
CV: Coefficient of variation

## References

[1] Hogg JC, Chu F, Utokaparch S, Woods R, Elliott WM, Buzatu L, et al. The nature of small-airway obstruction in chronic obstructive pulmonary disease. N Engl J Med. 2004;350:2645–53. 15215480 10.1056/NEJMoa032158

[2] Esther CR Jr, O’Neal WK, Anderson WH, Kesimer M, Ceppe A, Doerschuk CM, et al. Identification of sputum biomarkers predictive of pulmonary exacerbations in COPD. Chest. 2022;161:1239–49. 34801592 10.1016/j.chest.2021.10.049 PMC9131049

[3] Sagel SD, Chmiel JF, Konstan MW. Sputum biomarkers of inflammation in cystic fibrosis lung disease. Proc Am Thorac Soc. 2007;4:406–17. 17652508 10.1513/pats.200703-044BR PMC2647605

[4] Stockley RA, Halpin DMG, Celli BR, Singh D. Chronic obstructive pulmonary disease biomarkers and their interpretation. Am J Respir Crit Care Med. 2019;199:1195–204. 30592902 10.1164/rccm.201810-1860SO

[5] Gromelsky Ljungcrantz E, Askman S, Sjövall F, Paulsson M. Biomarkers in lower respiratory tract samples in the diagnosis of ventilator-associated pneumonia: a systematic review. Eur Respir Rev. 2025;34:240229. 40306955 10.1183/16000617.0229-2024 PMC12041932

[6] Dale I, Brandtzaeg P, Fagerhol MK, Scott H. Distribution of a new myelomonocytic antigen (L1) in human peripheral blood leukocytes. Immunofluorescence and immunoperoxidase staining features in comparison with lysozyme and lactoferrin. Am J Clin Pathol. 1985;84:24–34. 2409791 10.1093/ajcp/84.1.24

[7] Jukic A, Bakiri L, Wagner EF, Tilg H, Adolph TE. Calprotectin: from biomarker to biological function. Gut. 2021;70:1978–88. 34145045 10.1136/gutjnl-2021-324855 PMC8458070

[8] Polakowska M, Steczkiewicz K, Szczepanowski RH, Wysłouch-Cieszyńska A. Toward an understanding of the conformational plasticity of S100A8 and S100A9 Ca2+-binding proteins. J Biol Chem. 2023;299:102952. 36731796 10.1016/j.jbc.2023.102952 PMC10124908

[9] Wang S, Song R, Wang Z, Jing Z, Wang S, Ma J. S100A8/A9 in Inflammation. Front Immunol. 2018;9:1298. 29942307 10.3389/fimmu.2018.01298 PMC6004386

[10] Gao RY, Jia HM, Han YZ, Qian BS, You P, Zhang XK, et al. Calprotectin as a diagnostic marker for sepsis: a meta-analysis. Front Cell Infect Microbiol. 2022;12:1045636. 36519133 10.3389/fcimb.2022.1045636 PMC9742445

[11] Jarlborg M, Courvoisier DS, Lamacchia C, Martinez Prat L, Mahler M, Bentow C, et al. Serum calprotectin: a promising biomarker in rheumatoid arthritis and axial spondyloarthritis. Arthritis Res Ther. 2020;22:105. 32375861 10.1186/s13075-020-02190-3 PMC7201559

[12] Gray RD, Imrie M, Boyd AC, Porteous D, Innes JA, Greening AP. Sputum and serum calprotectin are useful biomarkers during CF exacerbation. J Cyst Fibros. 2010;9:193–8. 20299288 10.1016/j.jcf.2010.01.005

[13] Lee TH, Jang AS, Park JS, Kim TH, Choi YS, Shin HR, et al. Elevation of S100 calcium binding protein A9 in sputum of neutrophilic inflammation in severe uncontrolled asthma. Ann Allergy Asthma Immunol. 2013;111:268–75. 24054362 10.1016/j.anai.2013.06.028

[14] Huang X, Tan X, Liang Y, Hou C, Qu D, Li M, Huang Q, et al. Differential DAMP release was observed in the sputum of COPD, asthma and asthma-COPD overlap (ACO) patients. Sci Rep. 2019;9:19241. 31848359 10.1038/s41598-019-55502-2 PMC6917785

[15] Gray RD, MacGregor G, Noble D, Imrie M, Dewar M, Boyd AC, et al. Sputum proteomics in inflammatory and suppurative respiratory diseases. Am J Respir Crit Care Med. 2008;178:444–52. 18565957 10.1164/rccm.200703-409OC PMC2643212

[16] Mulvanny A, Pattwell C, Beech A, Southworth T, Singh D. Validation of sputum biomarker immunoassays and cytokine expression profiles in COPD. Biomedicines. 2022;10:1949. 36009496 10.3390/biomedicines10081949 PMC9405928

[17] IFU Gentian Calprotectin Reagent. REF1201, 7704–ven04.

[18] Aaron SD, Vandemheen KL, Ramsay T, Zhang C, Avnur Z, Nikolcheva T, Quinn A. Multi analyte profiling and variability of inflammatory markers in blood and induced sputum in patients with stable COPD. Respir Res. 2010;11:41. 20412595 10.1186/1465-9921-11-41 PMC2874769

[19] Valentin MA, Ma S, Zhao A, Legay F, Avrameas A. Validation of immunoassay for protein biomarkers: bioanalytical study plan implementation to support pre-clinical and clinical studies. J Pharm Biomed Anal. 2011;55:869–77. 21530130 10.1016/j.jpba.2011.03.033

[20] Cornes M, Simundic AM, Cadamuro J, Costelloe SJ, Baird G, Kristensen GBB, et al. CRESS checklist for reporting stability studies: on behalf of the European Federation of Clinical Chemistry and Laboratory Medicine (EFLM) Working Group for the Preanalytical Phase (WG-PRE). Clin Chem Lab Med. 2020;59:59–69. 32710715 10.1515/cclm-2020-0061

